# Borrelia multiplex: a bead-based multiplex assay for the simultaneous detection of *Borrelia* specific IgG/IgM class antibodies

**DOI:** 10.1186/s12879-022-07863-9

**Published:** 2022-11-17

**Authors:** Julia Häring, Max J. Hassenstein, Matthias Becker, Julia Ortmann, Daniel Junker, André Karch, Klaus Berger, Tatia Tchitchagua, Olaf Leschnik, Manuela Harries, Daniela Gornyk, Pilar Hernández, Berit Lange, Stefanie Castell, Gérard Krause, Alex Dulovic, Monika Strengert, Nicole Schneiderhan-Marra

**Affiliations:** 1 NMI Natural and Medical Sciences Institute at the University of Tübingen, Markwiesenstrasse 55, 72770 Reutlingen, Germany; 2 Department of Epidemiology, Helmholtz Centre for Infection Research, Brunswick, Germany; 3 Institute of Epidemiology and Social Medicine, University of Münster, Münster, Germany; 4 Department of Neurology, Sächsisches Krankenhaus Rodewisch, Rodewisch, Germany

**Keywords:** Borrelia, Lyme Disease, Lyme borreliosis, Multiplex, Immunoassay, Serology

## Abstract

**Background:**

Lyme borreliosis (LB) is the most common tick-borne infectious disease in the northern hemisphere. The diagnosis of LB is usually made by clinical symptoms and subsequently supported by serology. In Europe, a two-step testing consisting of an enzyme-linked immunosorbent assay (ELISA) and an immunoblot is recommended. However, due to the low sensitivity of the currently available tests, antibody detection is sometimes inaccurate, especially in the early phase of infection, leading to underdiagnoses.

**Methods:**

To improve upon *Borrelia* diagnostics, we developed a multiplex *Borrelia* immunoassay (Borrelia multiplex), which utilizes the new INTELLIFLEX platform, enabling the simultaneous dual detection of IgG and IgM antibodies, saving further time and reducing the biosample material requirement. In order to enable correct classification, the Borrelia multiplex contains eight antigens from the five human pathogenic *Borrelia* species known in Europe. Six antigens are known to mainly induce an IgG response and two antigens are predominant for an IgM response.

**Results:**

To validate the assay, we compared the Borrelia multiplex to a commercial bead-based immunoassay resulting in an overall assay sensitivity of 93.7% (95% CI 84.8–97.5%) and a specificity of 96.5% (95%CI 93.5–98.1%). To confirm the calculated sensitivity and specificity, a comparison with a conventional 2-step diagnostics was performed. With this comparison, we obtained a sensitivity of 95.2% (95% CI 84.2–99.2%) and a specificity of 93.0% (95% CI 90.6–94.7%).

**Conclusion:**

Borrelia multiplex is a highly reproducible cost- and time-effective assay that enables the profiling of antibodies against several individual antigens simultaneously.

**Supplementary Information:**

The online version contains supplementary material available at 10.1186/s12879-022-07863-9.

## Background

Lyme borreliosis (LB) is the most common tick-borne infectious disease in the moderate climates of the northern hemisphere [1, 2]. It is caused by species of the *Borrelia burgdorferi* sensu lato complex [3], which contains more than 15 different species of which at least five are known to be pathogenic to humans (*B. burgdorferi* sensu stricto (s.s.), *B. afzelii*, *B. garinii*, *B. spielmanii* and *B. bavariensis* [4]). Geographical distribution of the individual species varies, with only *B. burgdorferi* s.s. found in North America and *B. afzelii* and *B. garinii* as most common species in Europe. *B. spielmanii* and *B. bavariensis* (formerly *B. garinii* OspA type 4) were discovered later and are also found in Europe [1, 4–6]. *Borrelia* are transmitted to humans by various tick species of the genus *Ixodes*, with bacterial transmission occurring within the first 24 h of the tick bite [1, 2, 5–8].

Approximately 5% of individuals who are bitten by an infected tick will become infected, with manifest LB developing in 2% of individuals [4]. However, a comprehensive prediction of the annual new LB cases in Germany is difficult (e.g. due to the lack of a nationwide notification system) [4, 9]. One study based on data collected in 9 of 16 federal states reported a total of 56,446 new cases between 2013 and 2017 [9], while another from 2007/2008 assumes 214,000 new cases annually based on billing data from a German health insurance company [10]. According to the current care atlas from 2021, more than 300,000 patients are diagnosed with LB per year (based on nationwide billing data from public health insurances from 2010 to 2019) [11].

The diagnosis of LB is mainly based on clinical symptoms. An erythema migrans (EM) is considered a clear sign of LB [1, 3, 12–14] while the occurrence of a lymphocytoma, an acrodermatitis chronica atrophicans (ACA) or a meningoradiculoneuritis (Garin-Bujadoux-Bannwarth syndrome) also strongly indicates an infection [1, 13]. Due to the unspecific nature of the majority of symptoms, serological diagnosis is necessary for all manifestations except EM to support clinical diagnosis [1, 5, 13, 15]. Currently, two-tier testing consisting of an enzyme-linked immunosorbent assay (ELISA) and an immunoblot is recommended in various guidelines on LB in Europe [16–18]. The ELISA is used as a screening test and usually has a higher sensitivity than specificity. In the case of a positive or uncertain result, an immunoblot, a qualitative test with almost 100% specificity, is carried out for confirmation [2, 4, 19, 20]. However, this approach is considered imperfect, especially in the early phase of infection, due to problems with the sensitivity of the serological tests as a result of low antibody titers [10, 21, 22].

A promising and more sensitive approach might be a bead-based multiplex immunoassay that in contrast to a traditional ELISA, allows the distinct detection of both the IgG and the IgM response against several *Borrelia* antigens [23]. Such bead-based multiplex assays have been developed for a range of other pathogens, with the use of multiple antigens enabling increased sensitivity and specificity [24–26]. To improve LB diagnostics, we developed “Borrelia multiplex”, a multiplex immunoassay for the detection of specific IgG/IgM antibodies against eight different *Borrelia* antigens from the five human pathogenic *Borrelia* species in Europe. The assay is capable of being measured on both FLEXMAP 3D and INTELLIFLEX platform. While with the FLEXMAP 3D platform only one antibody class can be detected per well (single antibody detection), the INTELLIFLEX platform enables the simultaneous analysis of IgG and IgM (dual antibody detection). Following technical and clinical assay validation to standard EMA bioanalytical guidelines, we compared Borrelia multiplex performance to commercially available *Borrelia* tests. Finally, we analyzed serum samples from a German serological survey (MuSPAD study), providing information on the seropositivity of LB across Germany.

## Materials and methods

### *Borrelia* antigens

In total, eight recombinant antigens of all five human pathogenic species in Europe (*B. burgdorferi* s.s., *B. spielmanii*, *B. bavariensis*, *B. garinii* and *B. afzelii*) were used. The antigens were selected following a literature research on currently available LB diagnostics and are listed together with a UniProt reference in Additional file 1: Table S1. All used antigens are full-length and therefore include the immunodominant regions. Six antigens mainly induce an IgG response and two antigens an IgM response. The antigens were produced by the company tgcBIOMICS GmbH (Bingen, Germany) within the framework of a joint ZIM project of the BMWi (FKZ: ZF4585502AJ8) and can be obtained on request.

### Sample collection for assay validation

For the technical assay validation, eight *Borrelia* positive and four negative serum samples were used. Three positive samples and the negative samples were obtained from Central BioHub (Henningsdorf, Germany). The positive samples have been previously analyzed with the SERION ELISA classic Borrelia burgdorferi IgG/IgM (Virion\Serion, Würzburg, Germany) or the LIAISON Borrelia IgG/IgM Quant (DiaSorin, Saluggia, Italy). The negative samples were self-reported healthy. Five positive serum samples were provided by the Department of Neurology, Sächsisches Krankenhaus Rodewisch (Rodewisch, Germany). These samples were previously analyzed with an Anti-Borrelia-plus-VlsE-ELISA (IgG) and an Anti-Borrelia-ELISA (IgM) from Euroimmun (Lübeck, Germany).

For the clinical assay validation, 341 serum samples from the MEMO study (Memory and Morbidity in Augsburg Elderly) were used. The study was conducted in 1997/98 as a follow-up of the WHO MONICA Survey S2, Germany in 1989/90 and contains people of 65 years or older on 1st October 1997 who were living in Augsburg (Germany) [27]. The samples have been previously tested for LB as part of a study evaluating the mortality impact of seropositivity to pathogens associated with chronic infections in the elderly [28]. Ethics approval for the MEMO study was granted by the ethics committee of the University of Münster, Germany.

### MuSPAD sample collection

1555 serum samples from the MuSPAD study (Multilocal and Serial Prevalence Study on Antibodies against SARS-CoV-2 in Germany) [29] were analyzed for an IgG/IgM immune response against *Borrelia* antigens. MuSPAD was a serial cross-sectional SARS-CoV-2 seroprevalence study, with samples collected from July 2020 to August 2021 from eight different collection sites in Germany (Freiburg, Reutlingen, Aachen, Chemnitz, Hannover, Magdeburg, Osnabrück and Greifswald). Samples used in this study were from all locations except Hannover. All study participants were 18 years or older [29]. Approval for the study was granted by the Ethics Committee of the Hannover Medical School, Germany (9086_BO_S_2020).

### Antigen coupling

All *Borrelia* antigens were covalently coupled to spectrally distinct populations of carboxylated paramagnetic beads (MagPlex Microspheres, Luminex Corporation) using 1-ethyl-3-(3-dimethylaminopropyl)carbodiimide (EDC)/sulfo-*N*-hydroxysuccimide (sNHS) chemistry. The coupling was performed at room temperature with a magnetic particle processor (KingFisher 96, Thermo Fisher Scientific) and is based on the recommended procedure in the Luminex xMAP Cookbook [30].

Uncoupled beads were vortexed thoroughly and sonicated for 3 min. 1 mL of each individual bead population (1.25 × 10^7^ beads) was pipetted with 83 µL of 0.065% (v/v) Triton X-100 into different wells of a 96 deep well plate. The plate was placed in the particle processor and the coupling was started. Firstly, the beads were washed twice with 500 µL activation buffer (100 mM Na_2_HPO_4_, pH 6.2 + 0.005% (v/v) Triton X-100) and then activated for 20 min in 300 µL of 5 mg/mL EDC and 5 mg/mL sNHS in activation buffer. Subsequently, beads were washed again using 500 µL of coupling buffer (50 mM MES, pH 5.0 + 0.005% (v/v) Triton X-100) and incubated for 2 h with the antigens in coupling buffer. Depending on the antigen, a concentration of 0.50–10.0 µg antigen/10^6^ beads was used (Additional file 1: Table S1). The optimal concentration for coupling was determined as part of a pilot study (data not shown). After washing twice with wash buffer (1 × PBS, pH 7.4 + 0.005% (v/v) Triton X-100), the beads were resuspended in 1 mL storage buffer (1 × PBS, pH 7.4 + 1% (w/v) BSA + 0.05% (v/v) ProClin300). For subsequent use of the beads in the Borrelia multiplex, a bead mix with 1.0 × 10^6^ beads/mL per bead population was prepared. The beads were stored at 4 °C until used.

### Luminex platforms

Two different Luminex platforms were used—FLEXMAP 3D and INTELLIFLEX. The FLEXMAP 3D platform was used for the development and technical validation of the assay. Afterwards, the assay was transferred to the latest Luminex platform—INTELLIFLEX. While FLEXMAP 3D has two lasers (one for fluorescent beads and one for PE detection system), INTELLIFLEX has an additional laser enabling dual reporting. In this case, the INTELLIFLEX was used to allow parallel detection of two antibody classes within one well.

### Borrelia multiplex

#### Borrelia multiplex on FLEXMAP 3D platform

For the detection of *Borrelia* specific IgG/IgM antibodies, eight different *Borrelia* antigens were coupled to different bead populations as described above. The prepared bead mix was diluted 1:25 in assay buffer. The samples to be measured were diluted 1:200 in assay buffer and then incubated with the diluted bead mix in a 1:1 ratio (25 µL beads/sample, final sample dilution of 1:400) in a 96 half well plate (Corning, Cat# 3690). The assay plate was then incubated for 2 h at 21 °C and 750 rpm using a plate shaker (ThermoMixer C, Eppendorf). Afterwards, the beads were washed three times with 100 µL wash buffer (1 × PBS + 0.05% (v/v) Tween20) to remove unbound antibodies and resuspended in 100 µL of the same buffer using a microplate washer (BioTek MultiFlo FX, BioTek Instruments). Following the washing step, the beads were divided into two 96 half well plates. One plate was used for IgG detection, the other plate for IgM detection. IgG detection was done using a PE conjugated goat anti-human IgG antibody (Dianova, Cat# 109-116-098) at a concentration of 3 µg/mL. For IgM detection, a donkey anti-human IgM antibody (Dianova, Cat# 709-116-073) at 5 µg/mL was used. Per well, 30 µL of the corresponding antibody was pipetted and the beads were incubated again for 45 min at 21 °C and 750 rpm. To remove unbound detection antibodies, the beads were washed three times and resuspended in 100 µL wash buffer on plate shaker (21 °C, 1000 rpm for 3 min) before measurement. The measurement was carried out on a FLEXMAP 3D instrument. Settings were as followed: Uptake volume 80 µL, Count: 50/bead population, Timeout: 60 s, Gating: 7.500–15.000. Median Fluorescence Intensity (MFI) was measured. To monitor assay stability, quality control (QC) samples were included in each run. QC samples were prepared by diluting serum 200-fold in assay buffer. In addition, control beads were used to check detection system loading as well as sample loading for each well. A minimum bead threshold of 35 was applied to ensure accurate MFI calculation.

#### Borrelia multiplex on INTELLIFLEX platform

For the dual detection of *Borrelia* specific IgG and IgM antibodies on the INTELLIFLEX platform, the bead mix was diluted 1:50 in assay buffer. Samples were prepared as for the measurement on the FLEXMAP 3D platform and incubated with the beads for 2 h at 21 °C and 750 rpm on a plate shaker. Afterwards, the beads were washed three times with 100 µL wash buffer, but not resuspended. Instead of splitting the plate, detection systems were added directly. For IgG detection, a biotinylated goat anti-human IgG antibody (Dianova, Cat# 109-066-098) was used at a concentration of 1 µg/mL. IgM detection was performed using the donkey anti-human IgM antibody (5 µg/mL) already used on the FLEXMAP 3D platform. For simultaneous IgG/IgM detection, a mix of both antibodies was prepared and 30 µL per well were added to the beads. This was followed by an incubation at 21 °C and 750 rpm for 45 min. After washing three times with 100 µL wash buffer, Brilliant Violet 421 labelled streptavidin (BD Biosciences, Cat# 563259) for IgG detection was added to the beads at a concentration of 0.2 µg/mL. 30 µL were pipetted per well. A final incubation was performed at 21 °C and 750 rpm for 30 min. Then beads were washed again three times with 100 µL wash buffer and resuspended in 100 µL. After shaking the beads at 21 °C and 1000 rpm for 3 min, the measurement was performed with the same settings as for the FLEXMAP 3D platform.

#### Automated assay processing

To enable high-throughput screening, we semi-automated assay processing using a pipetting robot (Beckman Coulter i7, Beckman Coulter). The processing followed the same procedure as for IgG/IgM detection on the INTELLIFLEX platform. Instead of a 96 half well plate, a 384 well plate was used with the same volumes. Plate washing was done with a BioTek 405 TS plate washer (BioTek Instruments). Furthermore, plates were shaken at 1450 rpm instead of 750 rpm during the incubation times. Before measuring on the INTELLIFLEX instrument (Uptake volume 60 µL, Count: 40/bead population, Time out: 80 s, Gating 7.500–15.000), plates were shaken for 5 min at 21 °C and 1800 rpm.

### Technical assay validation

Technical validation of the Borrelia multiplex was carried out according to the "Guideline on bioanalytical method validation" of the European Medicines Agency (EMA) [31]. Intra- and inter-assay precision as well as the Limit of Detection (LOD) and the dilution linearity were considered. For the determination of the intra-assay precision, three serum samples were measured in 12 replicates on a 96 half well plate. Inter-assay precision was assessed by measuring QC samples. Measurements for the inter-assay precision were made in triplicates over five independent runs (FLEXMAP 3D platform) or in duplicates over seven runs (INTELLIFLEX platform). For LOD determination, assay buffer was measured without sample in 21 replicates. The LOD was calculated as the mean MFI + 3 × standard deviation. Data for intra- and inter-assay precision and LOD determination can be found in Additional file 1: Table S2. To investigate dilution linearity two serum samples were measured in a serial dilution series (1:100–1:12,800) in triplicates (Additional file 1: Fig. S1).

### Clinical assay validation

A clinical validation of the Borrelia multiplex was carried out in order to establish MFI cut-offs for the subsequent sample classification. For this purpose, the 341 samples of the MEMO study were measured in single replicates with the Borrelia multiplex and a commercial bead-based immunoassay from Mikrogen (see”Commercial *Borrelia* tests”). Based on the result of the commercial immunoassay, the measured samples were divided into *Borrelia* IgG/IgM negative or positive. MFI cut-off values were determined using ROC analysis (Additional file 1: Fig. S2). For the six IgG dominant antigens, cut-offs were determined by looking at the MFI signals of the IgG detection. For the two IgM dominant antigens, MFI signals of the IgM detection were used. The values were set to achieve a specificity of 95% or higher for each antigen, with different cut-offs tested in pilot trials to obtain the highest possible sensitivity combining all antigens. After the cut-off determination, three cut-off (CO) samples were generated through combining ten different positive sera as described in ref. [32]. Briefly, sera with high and low MFI signals were measured in a linear dilution series and the cut-off range for each antigen was defined. For each antigen, a fit was performed for the dilution series of each serum, allowing estimation of their behavior when combined. From there, sample pools were generated by combining sera in a way that the corresponding MFI signal would meet the defined cut-off. CO samples cover both IgM and IgG.

For each generated CO sample, MFI signals were achieved that were within the range of the corresponding cut-off for at least one antigen. CO sample 1 covered the cut-off of the BmpA–PKo. CO sample 2 covered the cut-offs of p83/100–PKo and OspC–A14S. The cut-offs of the remaining antigens were covered by the CO sample 3. The CO samples were included in each measurement to avoid an impact of plate fluctuations on the sample classification.

### Sample classification

**Fig. 1:**
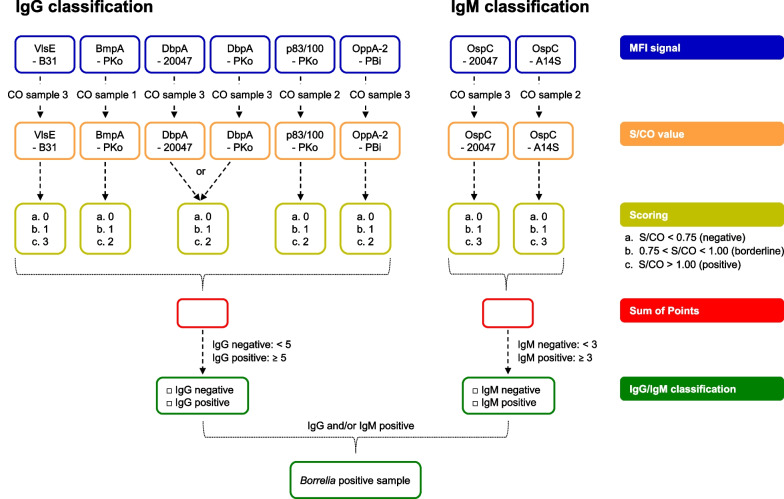
Sample classification of Borrelia multiplex. Sample classification was done as follows. In the first step the MFI signal of each antigen was divided by the MFI signal of the corresponding CO samples. The obtained S/CO values were then scored in a point system for each antigen. For BmpA, DbpA, p83/100 and OppA-2, values from 0 to 2 were assigned. For VIsE and OspC, values from 0 to 3 were used. For DbpA, the antigens were considered a pair, with only the highest value used. For each antigen, samples with a S/CO ratio < 0.75 received 0 points, samples with a S/CO ratio between 0.75 and 1.00, received 1 point and samples with a S/CO ratio > 1.00 received 2 or 3 points, depending on the antigen. After the classification of all antigens, IgG and IgM scores were generated (“Sum of Points”). For IgG, a score ≥ 5 results in a sample classification as IgG positive. For IgM, a score ≥ 3 results in a sample classification as IgM positive. If either antibody class is positive, than the entire sample was classified as *Borrelia* positive

An overview of the classification process is given in Fig. 1. For each antigen, the MFI signals of the serum (S) were divided by the MFI signal of the corresponding CO sample, giving S/CO values for each antigen. The S/CO values were then scored in a point system for each antigen. S/CO values were divided into three categories: negative (S/CO < 0.75), borderline (S/CO between 0.75 and 1.00) and positive (S/CO > 1.00). Negative values indicate that no antibody response to the antigen could be detected, whereas borderline values indicate that while a response may be present, it is not strong enough to directly classify as positive. For each antigen, negative values received 0 points, borderline values 1 point, and positive values 2 or 3 points depending upon the antigen. BmpA–PKo, DbpA–20047/PKo, p83/100–PKo, and OppA-2–PBi all received 2 points, as while these antigens are known to be specific for a *Borrelia* infection, an antibody response doesn’t have to occur. VlsE–B31, OspC–A14S and OspC–20047 were all assigned 3 points, as these antigens are the most important IgG and IgM dominant antigens, and their presence strongly suggests a *Borrelia* infection. All antigens received their own score for each sample, with the exception of the DbpA antigens, who are combined together in a pair with only the highest value used. For the borderline value different scorings were tested. The optimal value was chosen by ROC analysis (Additional file 1: Fig. S3A–C). For each sample, a Sum of Points was then calculated for IgG and IgM individually, by adding together the values of all antigens used in each antibody class. For IgG, a sum of 5 or higher indicated IgG positivity, while for IgM, a sum of 3 or higher was used. Optimization of the Sum of Points value for classification was also performed (Additional file 1: Fig. S3D–E). For the overall sample classification, either IgG or IgM positivity was considered sufficient to classify a sample as *Borrelia* positive.

### Commercial *Borrelia* tests

In addition to the measurements with the Borrelia multiplex, several serum samples were analyzed with commercially available *Borrelia* tests. An overview of all analyses is given in Additional file 1: Fig. S4.

#### recomBead Borrelia IgG/IgM 2.0 (Mikrogen)

The recomBead Borrelia IgG/IgM 2.0 from Mikrogen (München, Germany) was used to analyze serum samples from the MEMO study. For the clinical validation, all 341 samples were measured. To determine the assay accuracy of the Borrelia multiplex a subset of 319 samples was measured due to plate capacities in the automated processing. After consultation with Mikrogen, measurements were performed on the FLEXMAP 3D platform. First, the assay plate was equilibrated with 50 µL buffer and the samples to be measured were diluted 1:51-fold. 50 µL of the pre-diluted samples were then incubated together with 50 µL of bead solution first for 1 min at 37 °C and 600 rpm, then for 19 min at 37 °C and 0 rpm on a plate shaker (ThermoMix C, Eppendorf). After incubation, the samples were washed five times with 200 µL buffer using the BioTek MultiFlo FX washer (BioTek Instruments). Afterwards, 50 µL conjugate solution (anti-human IgG or anti-human IgM) was pipetted per well and the plate was incubated again as already described above. A final washing step (3 × 200 µL buffer) followed and the beads were resuspended in 100 µL Sheath Fluid (Luminex Corporation, Cat# 40-50023). Before measuring, beads were shaken for 1 min at 600 rpm.

#### *Borrelia afzelii* + VlsE IgG Europe ELISA/*Borrelia afzelii* IgM ELISA + Borrelia Europe plus TpN17 LINE IgG/Borrelia Europe LINE IgM (Virotech diagnostics)

662 samples of the MuSPAD cohort were analyzed with the current recommended 2-step diagnostics. First samples were measured with the *Borrelia afzelii* + VlsE IgG Europe ELISA and the ***Borrelia afzelii*** IgM ELISA from Virotech Diagnostics (Dietzenbach, Germany). In case of a positive result, samples were analyzed again with the Borrelia Europe plus TpN17 LINE IgG or the Borrelia Europe LINE IgM (also Virotech Diagnostics). All measurements were performed by the alphaomega Laboratory (Leipzig, Germany), which has a DIN EN ISO/IEC 17025:2005 accreditation (accreditation number D-PL-18167-01).

### Data analysis

Data collection and assignment to metadata was done with Microsoft Excel 2016. GraphPad Prism (version 9.3.1) was used for the graphical representation and statistical analysis of the data. Correlation analyses were carried out according to Spearman (two-tailed, 95% confidence interval). The correlation coefficient (Spearman r) is given for all analyses. The 95% confidence intervals for the stated sensitivities and specificities were calculated according to Wilson/Brown. The Mann–Whitney test (two-tailed, 95% confidence interval) was used to evaluate the association between S/CO values of *Borrelia* negative and positive samples. Samples of the MuSPAD study were analyzed using Fisher's exact test (two-tailed, 95% confidence interval, calculation of 95% CI according to Koopman). P values were classified as follows: > 0.05 (ns), ≤ 0.05 (*), ≤ 0.01 (**), ≤ 0.001 (***). Seropositivity was calculated using crude proportions and 95% confidence intervals.

## Results

### Borrelia multiplex assay performance and accuracy

**Fig. 2:**
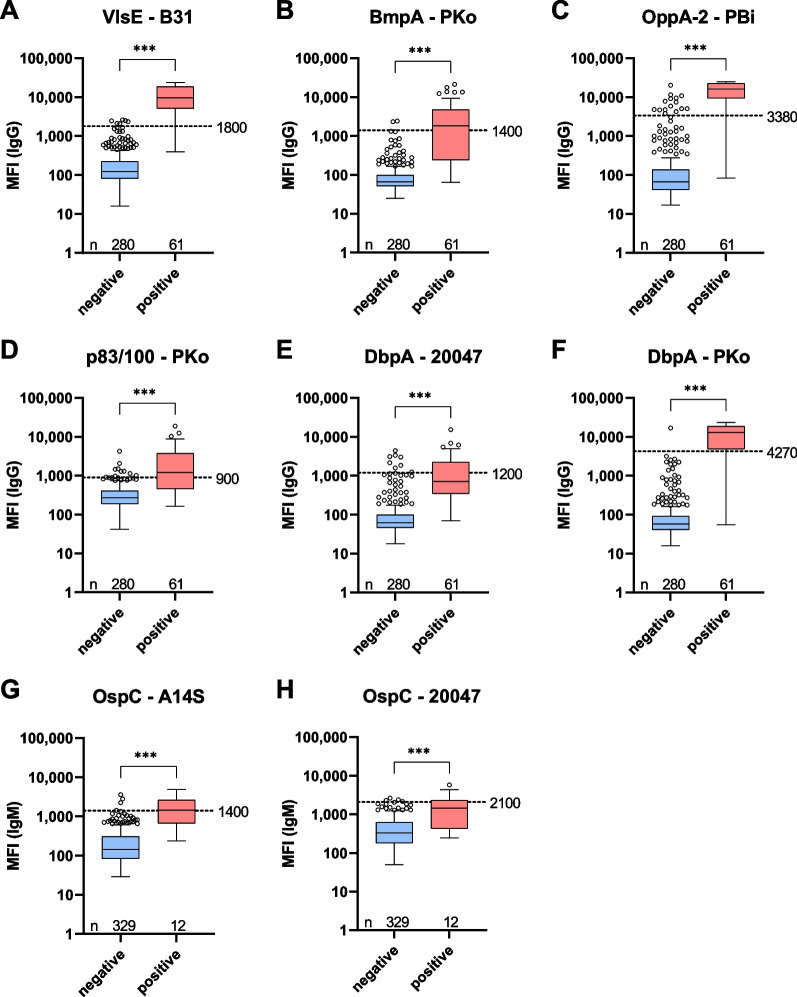
Antibody response in *Borrelia* negative and positive samples. 341 serum samples from the MEMO study were measured with the Borrelia multiplex and a commercial bead-based immunoassay (recomBead Borrelia IgG/IgM 2.0). Based on the result of the recomBead Borrelia samples were categorized in *Borrelia* negative and positive. The corresponding MFI signals of the Borrelia multiplex were plotted in a Box-Whisker plot. Boxes include the median and the 25th and 75th percentiles. Whiskers are limited to 1.5 times IQR. Outliers are shown as depicted circles. A dashed line (S/CO = 1.0) indicates the determined MFI cut-offs. For the antigens DbpA–ZS7, DbpA–A14S and DbpA–PBi no cut-offs were needed. For statistical analysis Mann–Whitney test (two-tailed) was used. P values were classified as follows: > 0.05 (ns), ≤ 0.05 (*), ≤ 0.01 (**), ≤ 0.001 (***). **A–F** Box-Whisker plots for IgG dominant antigens. **G**, **H** Box-Whisker plots for IgM dominant antigens

Using bead-based xMAP technology from the Luminex Corporation, we have developed a multiplex immunoassay for the detection of specific antibodies against eight *Borrelia* antigens from five different *Borrelia* species. 341 serum samples from the MEMO study were pretested with the recomBead Borrelia IgG/IgM 2.0, Mikrogen and categorized in *Borrelia* positive and negative samples. With the Borrelia multiplex, significant differences in the MFI signals between the categorized samples could be observed for all antigens (Fig. 2). However, the differences between the medians of negative and positive samples were smaller for some antigens than for others (e.g. OppA-2-PBi vs. OspC-20047) and outliers were observed for each antigen, indicating accurate sample classification can only be achieved by a combination of multiple antigens.

By analyzing the intra- and inter-assay precision during the technical assay validation, a high stability of the assay could be confirmed (Additional file 1: Table S2). The average coefficient of variation (%CV) for the intra-assay precision was less than 5% on both platforms. For the inter-assay precision, average %CVs of less than 10% were obtained for almost all antigens (except p83/100–PKo).

Using 319 of the samples pretested with the recomBead Borrelia IgG/IgM 2.0, an overall assay sensitivity of 93.7% (95%CI 84.8–97.5%) and an overall assay specificity of 96.5% (95%CI 93.5–98.1%) were calculated. Sensitivity and specificity for the different antibody classes are shown in Table 1. Calculated Cohen’s kappa coefficients can be found in Additional file 1: Table S3. With automated processing by pipetting robot, sensitivity decreased compared to the manual pipetting by approximately 10% (manual processing sensitivity 93.7%, automated processing sensitivity 84.1%) and a loss of IgG specificity (manual processing 96.5%, automated processing 90.2%) was observed. In general, there were larger differences in IgM than in IgG classification between the Borrelia multiplex and the recomBead Borrelia 2.0. With manual processing, six samples (1.88%) could be classified as IgM positive with the Borrelia multiplex, which were negative in the recomBead Borrelia IgM 2.0. Conversely, five samples (1.57%) were IgM positive with the recomBead Borrelia IgM 2.0, but negative in the Borrelia multiplex. In addition to the comparison of the Borrelia multiplex with the recomBead Borrelia IgG/IgM 2.0, our assay was compared with a conventional 2-step diagnostics (ELISA + Immunoblot). Calculated sensitivities and specificities are shown in Table 2. Here, as well, a lower sensitivity with automated processing could be determined while the specificity remained approximately the same. Only the sensitivity for IgM remained the same and was higher compared to the previously calculated sensitivity. However, there were also clear differences in the IgM classification between the Borrelia multiplex and the 2-step diagnostics. With manual processing, 38 samples (5.74%) could be classified as IgM positive with the Borrelia multiplex, which, in contrast, were classified as negative with the 2-step diagnostics. Conversely, only two samples (0.30%) were classified IgM positive with the 2-step diagnostics and were negative with the Borrelia multiplex. Altogether, the sensitivity and specificity calculated from the comparison with the recomBead Borrelia IgG/IgM 2.0 during clinical assay validation could be confirmed by the comparison with a conventional 2-step diagnostics.

**Table 1:** Sensitivity and specificity of Borrelia multiplex against the recomBead Borrelia IgG/IgM 2.0

Classification	Sensitivity		Specificity	
	Manual processing	Automated processing	Manual processing	Automated processing
IgG/IgM	recomBead positive: 63 Borrelia multiplex: positive consensus: 59 Sens.: 93.7% (84.8–97.5%) PPV: 86.8%	recomBead positive: 63 Borrelia multiplex: positive consensus: 53 Sens.: 84.1% (73.2–91.1%) PPV: 67.9%	recomBead negative: 256 Borrelia multiplex: negative consensus: 247 Spec.: 96.5% (93.5–98.1%) NPV: 98.4%	recomBead negative: 256 Borrelia multiplex: negative consensus: 231 Spec.: 90.2% (86.0–93.3%) NPV: 95.9%
IgG	recomBead positive: 58 Borrelia multiplex: positive consensus: 56 Sens.: 96.6% (88.3–99.4%) PPV: 93.3%	recomBead positive: 58 Borrelia multiplex: positive consensus: 50 Sens.: 86.2% (75.1–92.8%) PPV: 71.4%	recomBead negative: 261 Borrelia multiplex: negative consensus: 257 Spec.: 98.5% (96.1–99.4%) NPV: 99.2%	recomBead negative: 261 Borrelia multiplex: negative consensus: 241 Spec.: 92.3% (88.5–95.0%) NPV: 96.8%
IgM	recomBead positive: 11 Borrelia multiplex: positive consensus: 6 Sens.: 54.5% (28.0–78.7%) PPV: 50.0%	recomBead positive: 11 Borrelia multiplex: positive consensus: 5 Sens.: 45.5% (21.3–72.0%) PPV: 45.5%	recomBead negative: 308 Borrelia multiplex: negative consensus: 302 Spec.: 98.1% (95.8–99.1%) NPV: 98.4%	recomBead negative: 308 Borrelia multiplex: negative consensus: 302 Spec.: 98.1% (95.8–99.1%) NPV: 98.1%

Sensitivity and specificity were determined based on a comparison with the recomBead Borrelia IgG/IgM 2.0 (Mikrogen). The determination was carried out for IgG and IgM detection separately as well as for a combined analysis of both antibody classes. In total 319 samples of the MEMO study were analyzed. Number of positive and negative samples with both the commercial test and the Borrelia multiplex are indicated. Percentage indicates Borrelia multiplex performance (sensitivity/specificity) compared to the commercial test. 95% CI calculated by Wilson/Brown are shown. PPV—positive predictive value. NPV—negative predictive value.

**Table 2:** Sensitivity and specificity of Borrelia multiplex against a commercial 2-step diagnostics

Classification	Sensitivity		Specificity	
	Manual processing	Automated processing	Manual processing	Automated processing
IgG + IgM	2-Step testing positive: 42 Borrelia multiplex: positive consensus: 40 Sens.: 95.2% (84.2–99.2%) PPV: 47.6%	2-Step testing positive: 42 Borrelia multiplex: positive consensus: 36 Sens.: 85.7% (72.2–93.3%) PPV: 53.7%	2-Step testing negative: 620 Borrelia multiplex: negative consensus: 576 Spec.: 93.0% (90.6–94.7%) NPV: 99.7%	2-Step testing negative: 620 Borrelia multiplex: negative consensus: 589 Spec.: 95.0% (93.0–96.5%) NPV: 99.0%
IgG	2-Step testing positive: 39 Borrelia multiplex: positive consensus: 37 Sens.: 94.9% (83.1–99.1%) PPV: 80.4%	2-Step testing positive: 39 Borrelia multiplex: positive consensus: 32 Sens.: 82.1% (67.3–91.0%) PPV: 80.0%	2-Step testing negative: 623 Borrelia multiplex: negative consensus: 614 Spec.: 98.6% (97.3–99.2%) NPV: 99.7%	2-Step testing negative: 623 Borrelia multiplex: negative consensus: 615 Spec.: 98.7% (97.5–99.3%) NPV: 98.9%
IgM	2-Step testing positive: 7 Borrelia multiplex: positive consensus: 5 Sens.: 71.4% (35.9–94.9%) PPV: 11.6%	2-Step testing positive: 7 Borrelia multiplex: positive consensus: 5 Sens.: 71.4% (35.9–94.9%) PPV: 17.2%	2-Step testing negative: 655 Borrelia multiplex: negative consensus: 617 Spec.: 94.2% (92.1–95.7%) NPV: 99.7%	2-Step testing negative: 655 Borrelia multiplex: negative consensus: 631 Spec.: 96.3% (94.6–97.5%) NPV: 99.7%

Sensitivity and specificity were determined based on a comparison with a 2-step diagnostics consisting of an ELISA and an immunoblot (both Virotech Diagnostics). The determination was carried out for IgG and IgM detection separately as well as for a combined analysis of both antibody classes. In total 662 samples of the MuSPAD study were analyzed. Number of positive and negative samples with both the commercial 2-step testing and the Borrelia multiplex are indicated. Percentage indicates Borrelia multiplex performance (sensitivity/specificity) compared to the commercial 2-step testing. 95% CI calculated by Wilson/Brown are shown. PPV—positive predictive value. NPV—negative predictive value.

### Borrelia multiplex on different Luminex platforms

**Fig. 3:**
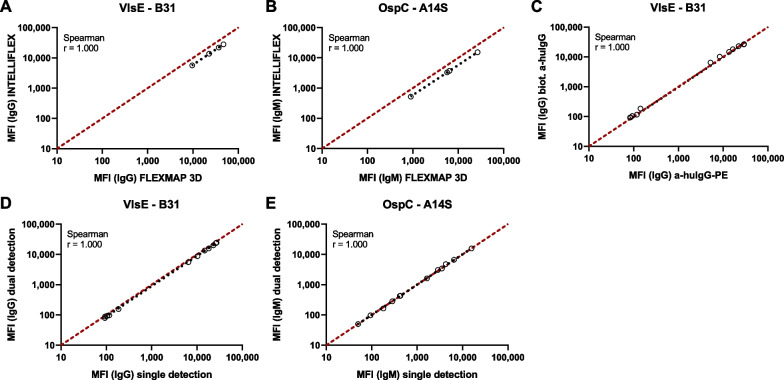
Adaption of Borrelia multiplex to the INTELLIFLEX platform. Adaption of the Borrelia multiplex to the new INTELLIFLEX platform was done in three steps. **A**, **B** 4 serum samples were measured for *Borrelia* specific IgG (**A**) and IgM (**B**) antibodies to compare the PE detection system on FLEXMAP 3D and INTELLIFLEX platform. **C** 12 serum samples were measured for IgG antibodies to compare a directly PE-labelled detection antibody with a biotinylated antibody in combination with BV421 labelled streptavidin. **D**, **E** 12 serum samples were measured for IgG (**D**) and IgM (**E**) antibodies separately (single detection) or in combination (dual detection). In each graph MFI signals were plotted against each other and analyzed by linear regression. A linear curve (x = y) shown as red dashed line indicates identical MFI signals for detection systems. Correlation analysis was performed after Spearman

The Borrelia multiplex can be used on two different Luminex platforms—FLEXMAP 3D and INTELLIFLEX. For measurements with the FLEXMAP 3D instrument, the *Borrelia* specific IgG or IgM antibodies were detected using phycoerythrin (PE) conjugated species-specific antibodies. In order to be able to detect IgG and IgM antibodies in parallel on the INTELLIFLEX platform, the conjugated fluorescent dye of one detection system had to be changed from PE to Brilliant Violet 421 (BV421). Before changing the detection system of an antibody class, the used PE systems were measured for a comparison of the MFI on a FLEXMAP 3D and an INTELLIFLEX instrument. For all antigens, there was a strong correlation between the MFI signals of both platforms (Fig. 3A, B; Additional file 1: Fig. S5). However, the MFI signals of the INTELLIFLEX measurement were on average 1.7-fold lower than the values of the FLEXMAP 3D measurement. As CO samples were included with each measurement, sample classification was unaffected. In a second experiment, the PE conjugated species-specific antibody for IgG detection was replaced by a biotinylated antibody paired with a BV421 labelled streptavidin. A comparison with the previous detection system showed a strong correlation of MFI signals (Fig. 3C; Additional file 1: Fig. S6). After adapting the IgG detection system, it was possible to detect both antibody classes in one run. The MFI signals of the dual detection correlated strongly with the MFI signals of the single detection (Fig. 3D, E; Additional file 1: Fig. S7).

### Assay automatization for large-scale screening

**Fig. 4:**
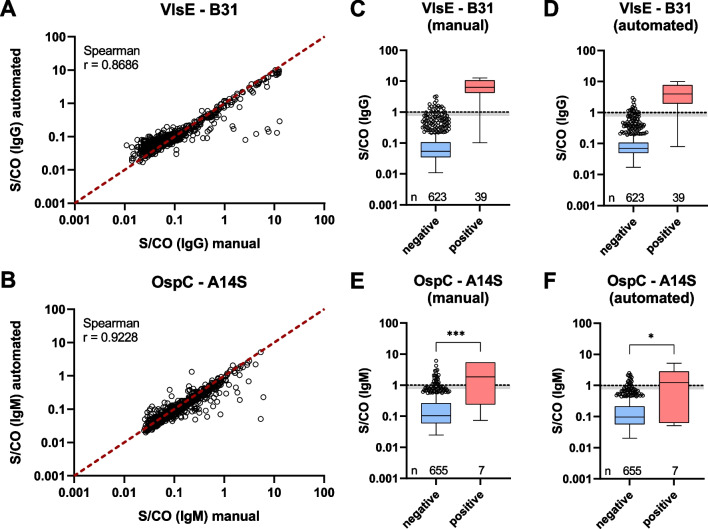
Manual and automated processing of Borrelia multiplex. 662 serum samples were measured with the Borrelia multiplex and a common 2-step diagnostics. For the Borrelia multiplex processing was either manually or by pipetting robot. **A,** **B** Correlation between manual and automated processing of Borrelia multiplex. S/CO values were plotted against each other. A red dashed line shows the curve for identical S/CO values. Correlation analysis was performed according to Spearman. **C–F** Box-Whisker plots of S/CO values for *Borrelia* negative and positive samples. Samples were categorized according to the result of the 2-step diagnostics. Boxes include the median and the 25th and 75th percentiles. Whiskers are limited to 1.5 times IQR. Outliers are shown as depicted circles. A dashed line (S/CO = 1.0) indicates the threshold between positive and negative signal. The grey area below the line gives the borderline area (0.75 < S/Co < 1.0). For statistical analysis Mann–Whitney test (two-tailed) was used. P values were classified as follows: > 0.05 (ns), ≤ 0.05 (*), ≤ 0.01 (**), ≤ 0.001 (***)

Since the developed assay was also to be used in large sample screenings, the assay was modified for automated processing using a pipetting robot (Beckman Coulter i7, Beckman Coulter). For high-throughput measurements, the plate format was changed to 384 well. For a comparison of both processing methods, 662 serum samples from the MuSPAD study were processed manually as well as automated and measured on the INTELLIFLEX platform. For almost all antigens, the comparison of the S/CO values (antigen MFI divided by MFI cut off) showed higher values for some samples when processed manually (Fig. 4A, B; Additional file 1: Fig. S8A–F). A categorization of the samples into *Borrelia* IgG/IgM negative and positive based on the result of the 2-step diagnostics (Fig. 4C–F; Additional file 1: Fig. S8G–R), indicated that the higher values were mainly found in positively categorized samples. However, sample classification remained consistent, with 652 samples classified equally for IgG (98.5%) and 646 samples classified equally for IgM (97.6%). The majority of samples with different classification was positive when processed manually, but negative when processed in an automated way.

### LB seropositivity across Germany

**Fig. 5:**
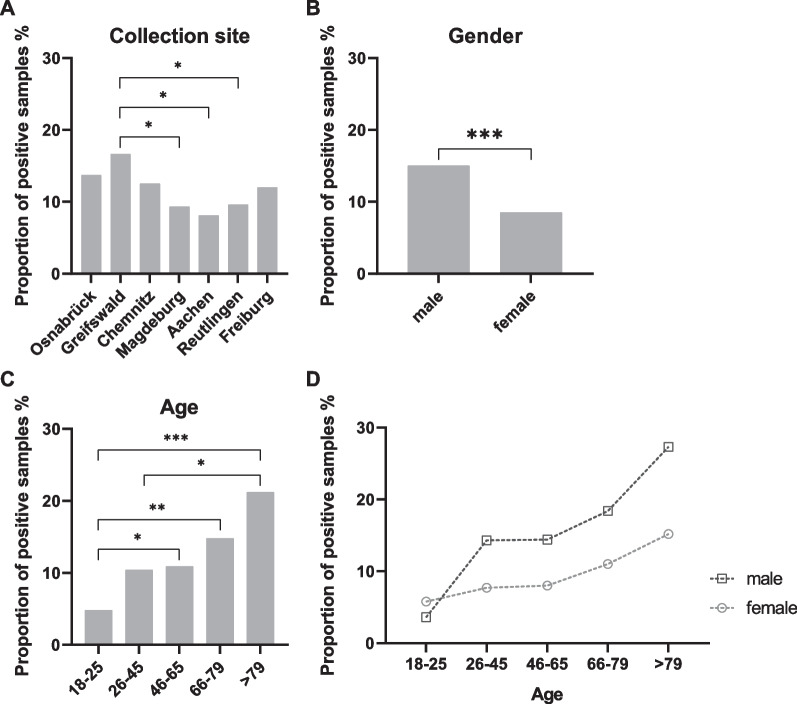
Epidemiological analysis of 1,555 serum samples from MuSPAD study. 1555 serum samples from the MuSPAD study were measured with the automated Borrelia multiplex and analyzed regarding collection site (**A**), gender (**B**) and age (**C**). Additionally, seropositivity of men and women was compared over all age groups (**D**). For statistical analysis Fisher’s exact test was used. P values were classified as follows: > 0.05 (ns), ≤ 0.05 (*), ≤ 0.01 (**), ≤ 0.001 (***)

Finally, 1,555 samples of a German serological survey (MuSPAD study) were measured with the developed automated Borrelia multiplex to obtain insights into the LB seropositivity across Germany. Of these samples, 115 samples were IgG positive (7.4%, 95%CI 6.2–8.8%) in the Borrelia multiplex. 73 samples were IgM positive (4.7%, 95%CI 3.8–5.9%). This resulted in an overall seropositivity (IgG and/or IgM positivity) of 11.4% (95%CI 10.0–13.1%, n = 178). The samples were collected at seven different sites in Germany. A comparison of the percentage of positive samples (IgG and/or IgM) at the respective collection sites is shown in Fig. 5A. Greifswald had the highest proportion of positive samples with 16.6% (95%CI 11.8–22.8%). In comparison, significantly lower seropositivity was found in Magdeburg (9.3%, 95%CI 6.1–14.0%), Aachen (8.1%, 95%CI 4.9–13.2%) and Reutlingen (9.6%, 95%CI 6.9–13.2%). For Osnabrück (13.7%, 95%CI 9.4–19.6%), Chemnitz (12.5%, 95%CI 8.4–18.2%) and Freiburg (12.0%, 95%CI 8.8–16.2%) no significant differences in seropositivity were found. Sample classification was further stratified by gender (Fig. 5B) and age (Fig. 5C), with almost twice as many positive samples (IgG and/or IgM) among males (15.0%, 95%CI 12.5–17.8%) as among females (8.5%, 95%CI 6.8–10.6%). Significant differences in the IgG and/or IgM positivity were also found regarding different age groups. The percentage of positive samples in the group of 18–25 year olds was 4.8% (95%CI 2.2–10.1%) and increased to 21.2% (95%CI 13.1–32.5%) in the group aged 79 years and above. In Fig. 5D, the proportion of positive samples in the different age groups was examined separately for men and women. Here, the proportion of positive samples was also higher among men than among women in almost all age groups. Only in the age group of 18–25 years, the proportion of positive samples was slightly higher among women. Absolute values and the corresponding percentages for the graphs are listed in Additional file 1: Table S4.

## Discussion

Serological analysis through detection of *Borrelia* specific IgG and IgM antibodies [23] in a two-step diagnostics is currently used to support a clinical LB diagnosis consistent with classical LB symptoms [1, 5, 13, 15]. However, seroconversion in an early infection can currently be detected in 20–50% of all patients [15]. Due to the Luminex xMAP platform utilized in our work, our developed Borrelia multiplex seems to be more sensitive in this infection stage. Compared to the classical two-step diagnostics, we identified 38 IgM positive samples more, suggesting increased sensitivity for early detection. However, the calculated IgM sensitivity does not come with high precision, which is due to the relatively small number of IgM positive samples. For this reason, caution should also be applied to the sensitivity calculated compared to the recomBead Borrelia IgM 2.0, a multiplex assay which also uses the xMAP technology with a similar antigen panel. Differences between the Borrelia multiplex and the recomBead Borrelia IgM 2.0 are likely due to different criteria for IgM classification of both assays. While only two OspC antigens are used for classification in the Borrelia multiplex, the recomBead Borrelia IgM 2.0 also includes the IgG dominant antigens of the assay. As a result, samples are classified as IgM positive even if they react only against several IgG dominant antigens of the assay. In the Borrelia multiplex, IgM sample classification is done by using only the IgM dominant OspC antigens. As consequence, fewer samples were classified as IgM positive and thus our Borrelia multiplex appeared less sensitive than it might be.

When examining the sensitivity and specificity of the Borrelia multiplex, it must be considered that they were calculated based on the comparison with another test, which is not the gold standard for LB diagnostics and has a certain inaccuracy by itself. Therefore, the assessment of the accuracy of the Borrelia multiplex is mainly an estimation. To further optimize the assay and determine the true sensitivity and specificity, a well-characterized sample set is required. This would also enable thorough evaluation against the commercially available tests, as they often lack standardization against a well-characterized sample set [14, 19, 22].

Antigen selection plays a major role in the development of a successful serological assay. For LB in Europe, heterogeneity between the different species must be considered. For this reason, our selected antigens had to fulfill two requirements. First, the antigenic diversity had to be covered. Secondly, no cross-reactions to other pathogens should occur [15, 23]. As a result, six antigens which are considered to be specific for *Borrelia burgdorferi* sensu lato [15, 33] were selected for IgG detection. The most important antigen is VlsE, which is considered strongly immunogenic [1] and triggers an IgG response even shortly after infection [15]. Analysis of amino acid sequence homology showed strong antigen heterogeneity [15]. However, since it has an immunodominant conserved epitope (C6 peptide) [15] and we could detect an immune response against the VlsE of the species *B. burgdorferi* in almost all samples, the use of one species was found to be sufficient. One species only was also chosen for the antigens BmpA, p83/100 and OppA-2, since low heterogeneity between the species was determined. The antigen DbpA, on the other hand, was used from all five species, as it is highly heterogeneous [12, 15] (approximate 45% amino acid sequence homology) and no conserved epitope is known. However, during the clinical assay validation, it became apparent that not all DbpA species are needed for a sample classification over several antigens. For the IgM detection, only the antigen OspC was used. This is an essential virulence factor for the dissemination [5, 6, 34] and is considered the most immunodominant antigen of the IgM response [15, 23]. It also exhibits strong heterogeneity, but like VlsE, it has an immunodominant conserved epitope (C10 peptide) [15]. Two species were sufficient for reliable IgM detection as an IgM response against at least three OspC species was observed in most positive samples of the pre-tests done during assay development. The antigens of the species *B. garinii* and *B. spielmanii* were chosen because they had the highest protein stability and the best reproducibility of coupling compared to the other species. OspC is not relevant for IgG detection, as *Borrelia* species regulate the expression shortly after infection in order to avoid a too strong immune response [6, 8, 35]. P41 is a common antigen utilized in other commercial assays for *Borrelia* detection. We initially tested an internal fragment of p41 during the early stages of assay development, however. However, this gave poor results and no difference in the MFI signals of negative and positive samples could be obtained. As p41 is a relatively unspecific antigen and cross-reactions with other pathogens may occur, we decided not to use this antigen. The different antigen compositions in LB tests are considered a general problem in diagnostics [19] and can lead to different test results. We show that correct sample classification can only be achieved through the combination of different antigens.

With the Borrelia multiplex, we have developed a robust assay which gives results in a reproducible way. The simple assay principle makes it possible to quickly modify the assay after the development and adapt it to different requirements. A special feature of the assay is the simultaneous detection of IgG and IgM antibodies. As shown when the assay was transferred from the FLEXMAP 3D to the INTELLIFLEX, the MFI signals of the single antibody detection correlated with the MFI signals of the simultaneous detection. Therefore, there was no cross-reaction between the used detection antibodies. A displacement of the weaker binding IgM antibodies by IgG could be almost excluded in preliminary tests with an RF absorbent. Otherwise, the samples would have to be pretreated before IgM detection using an RF absorbent, with which IgG antibodies can be bound and removed. A simultaneous IgG/IgM detection wouldn’t be possible in this case. When comparing between manual and automated processing of our assay, there were some differences in classification presumably due to more precise work during manual processing. With manual processing, special characteristics of a sample, such as lipaemia, can be considered, which is not possible with automated processing. However, since a different classification was found in less than 5% of samples, this problem is acceptable when screening large sample sets.

In Germany, an exact estimation of LB prevalence is difficult, in part due to the lack of compulsory notification in 7 out of 16 federal states [9]. Large longitudinal studies are therefore essential to obtain an accurate overview of seroconversion in the population. Due to its high-throughput automated format and thus the possibility of fast and efficient measurement of large sample sets, our Borrelia multiplex is an appropriate tool for such studies. To demonstrate this, we analyzed samples from MuSPAD, a serial cross-sectional population cohort.

Comparing seropositivity proportions between studies is difficult, as divergent classification algorithms significantly affect the reported seropositivity [36] and potentially more than double seropositivity. In addition, caution is advised when comparing assay-based differences in seropositivity, given the regional differences in infection risk [9, 37]. However, when comparing the seropositivity proportions of our study with regional proportions reported by two previous German cohorts [38], the results generally overlap regarding reported confidence intervals. We identified that LB seropositivity was twice as high in male study participants (15.0%, 95%CI 12.5–17.8%) than in female (8.5%, 95%CI 6.8–10.6%). This is in line with studies performed in Germany and Scandinavia [38–40]. We also observed an increase in anti-*Borrelia* antibodies with age, as seen in all previous studies among adults [9, 38–41]. Interestingly, no major differences in the geographical distribution of LB between the collection sites were found. Only for Greifswald (Mecklenburg Western Pomerania) a slightly higher seropositivity (16.6%, 95%CI 11.8—22.8%) could be detected, as previously seen by others [41].

## Conclusion

In summary, we successfully developed a robust multiplex assay for the analysis of the serostatus regarding a *Borrelia* infection. Due to its high-throughput automated format, it is perfectly suited for fast and efficient measurements of large sample sets. The simultaneous detection of IgG and IgM antibodies eliminates the need for duplicate testing, which saves time and money, but also reduces the consumption of limited biomaterial.

## Supplementary Information

**Additional** **file** **1:** **Table** **S1:** *Borrelia* Antigens used in the Borrelia multiplex. **Table S2**: Assay precision and LOD of the Borrelia multiplex on different Luminex platforms**.** . **Table** **S3:** Cohen’s kappa coefficients for the comparison of the Borrelia multiplex with commercial *Borrelia* test. **Table** **S4:** Serostatus of 1,555 serum samples from a German serological survey (MuSPAD study). **Figure** **S1:** Dilution linearity of Borrelia multiplex on different Luminex platforms.. **Figure** **S2:** ROC analysis for *Borrelia* specific IgG/IgM detection. **Figure** **S3:** Sample classification algorithms optimization. **Figure** **S4**: Sample analyses with commercial* Borrelia* tests. **Figure** **S5:** Comparison of PE detection system on FLEXMAP 3D and INTELLIFLEX platform. **Figure** **S6:** Comparison of IgG detection systems on INTELLIFLEX platform. **Figure** **S7:** Correlation between single detection and dual detection of IgG/IgM antibodies. **Figure** **S8:** Manual and automated processing of Borrelia multiplex.

## Data Availability

Data relating to the findings of this study are available from the corresponding author upon request. Source data are provided with this paper. The *Borrelia* antigens were provided by the company tgcBIOMICS GmbH (Bingen, Germany) and can be obtained on request.
