# Supplementary material for: Borrelia multiplex: a bead-based multiplex assay for the simultaneous detection of Borrelia specific IgG/IgM class antibodies
Source: BMC Infect Dis. 2022 Nov 17;22:859. doi: 10.1186/s12879-022-07863-9 (PMC9670078; doi:10.1186/s12879-022-07863-9)
Supplement: Supplementary file 1 — Additional file 1: Table S1: Borrelia Antigens used in the Borrelia multiplex. Table S2: Assay precision and LOD of the Borrelia multiplex on different Luminex platforms. . Table S3: Cohen’s kappa coefficients for the comparison of the Borrelia multiplex with commercial Borrelia test. Table S4: Serostatus of 1,555 serum samples from a German serological survey (MuSPAD study). Figure S1: Dilution linearity of Borrelia multiplex on different Luminex platforms.. Figure S2: ROC analysis for Borrelia specific IgG/IgM detection. Figure S3: Sample classification algorithms optimization. Figure S4: Sample analyses with commercial Borrelia tests. Figure S5: Comparison of PE detection system on FLEXMAP 3D and INTELLIFLEX platform. Figure S6: Comparison of IgG detection systems on INTELLIFLEX platform. Figure S7: Correlation between single detection and dual detection of IgG/IgM antibodies. Figure S8: Manual and automated processing of Borrelia multiplex. [file 12879_2022_7863_MOESM1_ESM.docx]

**Borrelia multiplex: A bead-based multiplex assay for the simultaneous detection of Borrelia specific IgG/IgM class antibodies**

Julia Häring^1^, Max J. Hassenstein^2^, Matthias Becker^1^, Julia Ortmann^2^, Daniel Junker^1^, André Karch^3^, Klaus Berger^3^, Tatia Tchitchagua^4^, Olaf Leschnik^4^, Manuela Harries^2^, Daniela Gornyk^2^, Pilar Hernández^2^, Berit Lange^2^, Stefanie Castell^2^, Gérard Krause^2^, Alex Dulovic^1,^*, Monika Strengert^2^, Nicole Schneiderhan-Marra^1,^*

Author Affiliations:

^1^ NMI Natural and Medical Sciences Institute at the University of Tübingen, Reutlingen, Germany

^2^ Department of Epidemiology, Helmholtz Centre for Infection Research, Braunschweig, Germany

^3^ Institute of Epidemiology and Social Medicine, University of Münster, Münster, Germany

^4^ Department of Neurology, Sächsisches Krankenhaus Rodewisch, Rodewisch, Germany

* Corresponding authors:

Dr. Alex Dulovic

Markwiesenstrasse 55, 72770 Reutlingen, Germany

Phone: 0049 7121 51530 580

Fax: 0049 7121 51530 16

E-Mail: Alex.Dulovic@nmi.de

Dr. Nicole Schneiderhan-Marra

Markwiesenstrasse 55, 72770 Reutlingen, Germany

Phone: 0049 7121 51530 815

Fax: 0049 7121 51530 16

E-Mail: Nicole.Schneiderhan@nmi.de

**Table S1: *Borrelia* Antigens used in the Borrelia multiplex.**

| **Antibody response** | **Antigen** | **Species** | **Strain** | **Stage of infection** | **UniProt**  **reference** | **Coupling Concentration**  **(µg / 10^6 beads)** |
| --- | --- | --- | --- | --- | --- | --- |
| IgG | VlsE - B31 | B. burgdorferi s.s | B31 | late | G5IXI6 | 1.25 |
|  | BmpA – Pko | B. afzelii | PKo | early / late | Q0SND0 | 1.25 |
|  | p83/100 – Pko | B. afzelii | PKo | late | A0A2S0X2C1 | 0.50 |
|  | OppA-2 – Pbi | B. bavariensis | PBi | late | A0A7I6GW20 | 1.25 |
|  | DbpA – 20047 | B. garinii | 20047 | late | Q9ZH83 | 10.00 |
|  | DbpA – Pko | B. afzelii | PKo | late | Q9ZH93 | 5.00 |
| IgM | OspC - A14S | B. spielmanii | A14S | early | C0RBX1 | 5.00 |
|  | OspC – 20047 | B. garinii | 20047 | early | B8F168 | 5.00 |

In total, eight *Borrelia* antigens of the five human pathogenic *Borrelia* species in Europe were used for the Borrelia multiplex. Antigens were produced by the company tgcBIOMICS GmbH (Bingen, Germany). For each antigen, a sequence identifier (UniProt reference) and the coupling concentration used is provided.

**Table S2: Assay precision and LOD of the Borrelia multiplex on different Luminex platforms.**

| **Antibody response** | **Antigen** | **FLEXMAP 3D** | | | **INTELLIFLEX** | | |
| --- | --- | --- | --- | --- | --- | --- | --- |
|  |  | **Intra-assay precision**  **(%CV)** | **Inter-assay precision**  **(%CV)** | **LOD**  **(MFI)** | **Intra-assay precision**  **(%CV)** | **Inter-assay precision**  **(%CV)** | **LOD**  **(MFI)** |
| IgG | VlsE - B31 | 1.3 | 3.9 | 29 | 1.9 | 5.1 | 8 |
|  | BmpA - PKo | 2.4 | 4.7 | 32 | 2.8 | 7.0 | 10 |
|  | p83/100 - PKo | 4.6 | 9.7 | 33 | 4.7 | 16.3 | 9 |
|  | OppA-2 - PBi | 1.3 | 3.4 | 31 | 2.0 | 4.5 | 9 |
|  | DbpA - 20047 | 3.2 | 4.4 | 37 | 2.8 | 6.4 | 8 |
|  | DbpA - PKo | 1.9 | 2.8 | 42 | 2.1 | 5.3 | 9 |
| IgM | OspC - 20047 | 1.7 | 1.9 | 28 | 2.4 | 3.9 | 8 |
|  | OspC - 20047 | 1.7 | 1.9 | 28 | 2.4 | 3.9 | 8 |

Assay precision (intra-inter assay precision) and the LOD (limit of detection) were assessed for each antigen on both Luminex platforms. For the intra-assay precision three serum samples were measured in 12 replicates. For the inter-assay precision on the FLEXMAP 3D platform three serum samples were measured in triplicates over five independent runs. On the INTELLIFLEX platform samples were measured in duplicates over seven independent runs. All %CVs (coefficients of variation) are shown as mean from the measured samples. For determination of the LOD, assay buffer was measured in 21 replicates. The LOD was calculated as the mean MFI + 3x standard deviation.

**Table S3: Cohen’s kappa coefficients for the comparison of the Borrelia multiplex with commercial *Borrelia* test.**

| **Classification** | **recomBead Borrelia IgG/IgM 2.0** | | **2-step diagnostics** | |
| --- | --- | --- | --- | --- |
|  | **manual processing** | **automated processing** | **manual processing** | **automated processing** |
| IgG/IgM | 0.875  (95%CI 0.809 - 0.941) | 0.682  (95%CI 0.585 - 0.779) | 0.601  (95%CI 0.499 - 0.704) | 0.632  (95%CI 0.523 - 0.740) |
| IgG | 0.938  (95%CI 0.888 - 0.987) | 0.727  (95%CI 0.632 - 0.822) | 0.862  (95%CI 0.781 - 0.942) | 0.798  (95%CI 0.699 - 0.897) |
| IgM | 0.504  (95%CI 0.249 - 0.758) | 0.435  (95%CI 0.135 - 0.700) | 0.185  (95%CI 0.041 - 0.330) | 0.265  (95%CI 0.075 - 0.455) |

The assay performance of the Borrelia multiplex was determined based on the comparison with the commercial recomBead Borrelia IgG/IgM 2.0 and a commercial 2-step diagnostics. The determination was carried out for IgG and IgM detection separately as well as for a combined analysis of both antibody classes. Cohen’s kappa coefficients were calculated with GraphPad Qick Calc (<https://www.graphpad.com/quickcalcs/kappa2/>, accessed 05.08.2022).

**Table S4: Serostatus of 1,555 serum samples from a German serological survey (MuSPAD study).**

| **Category** | | ***Borrelia* seronegative**  **n (%)** | ***Borrelia* seropositive**  **n (%)** |
| --- | --- | --- | --- |
| **Collection site**  n = 1555 | **Osnabrück**  n = 175 | 151 (86.3 %)  (95%CI 80.4 - 90.6 %) | 24 (13.7 %)  (95%CI 9.4 - 19.6 %) |
|  | **Greifswald**  n = 175 | 146 (83.4 %)  (95%CI 77.2 - 88.2 %) | 29 (16.6 %)  (95%CI 11.8 - 22.8 %) |
|  | **Chemnitz**  n = 176 | 154 (87.5 %)  (95%CI 81.8 - 91.6 %) | 22 (12.5 %)  (95%CI 8.4 - 18.2 %) |
|  | **Magdeburg**  n = 214 | 194 (90.7 %)  (95%CI 86.0 - 93.9 %) | 20 (9.3 %)  (95%CI 6.1 - 14.0 %) |
|  | **Aachen**  n = 172 | 158 (91.9 %)  (95%CI 86.8 - 95.1 %) | 14 (8.1 %)  (95%CI 4.9 - 13.2 %) |
|  | **Reutlingen**  n = 343 | 310 (90.4 %)  (95%CI 86.8 - 93.1 %) | 33 (9.6 %)  (95%CI 6.9 - 13.2 %) |
|  | **Freiburg**  n = 300 | 264 (88.0 %)  (95%CI 83.8 - 91.2 %) | 36 (12.0 %)  (95%CI 8.8 - 16.2 %) |
| **Gender**  n = 1555 | **male**  n = 708 | 602 (85.0 %)  (95%CI 82.2 - 87.5 %) | 106 (15.0 %)  (95%CI 12.5 - 17.8 %) |
|  | **female**  n = 847 | 775 (91.5 %)  (95%CI 89.4 - 93.2 %) | 72 (8.5 %)  (95%CI 6.8 - 10.6 %) |
| **Age**  n = 1555 | **18-25**  n = 125 | 119 (95.2 %)  (95%CI 90.0 - 97.8 %) | 6 (4.8 %)  (95%CI 2.2 – 10.1 %) |
|  | **26-45**  n = 450 | 403 (89.6 %)  (95%CI 86.4 - 92.1 %) | 47 (10.4 %)  (95%CI 7.9 - 13.6 %) |
|  | **46-65**  n = 617 | 550 (89.1 %)  (95%CI 86.4 - 91.4 %) | 67 (10.9 %)  (95%CI 8.6 - 13.6 %) |
|  | **66-79**  n = 297 | 253 (85.2 %)  (95%CI 80.7 - 88.8 %) | 44 (14.8 %)  (95%CI 11.2 - 19.3 %) |
|  | **>79**  n = 66 | 52 (78.8 %)  (95%CI 67.5 - 86.9 %) | 14 (21.2 %)  (95%CI 13.1 - 32.5 %) |

1,555 serum samples from a German serological survey (MuSPAD study) were measured with the automated Borrelia multiplex and analyzed regarding collection site, gender and age.

**Figure S1: Dilution linearity of Borrelia multiplex on different Luminex platforms.** Two serum samples were measured in different dilutions for *Borrelia* specific IgG **(A-F)** and IgM **(G and H)**. On the FLEXMAP 3D platform, both antibody classes were detected separately using a PE detection system. On the INTELLIFLEX platform, antibodies were detected in one run using a biotinylated antibody paired with a BV421 streptavidin for IgG detection. Each data point represents the mean MFI signal of triplicates with standard deviation (SD *) indicated by error bars. * Small SDs are not visible in this scale.

**Figure S2: ROC analysis for *Borrelia* specific IgG/IgM detection.** Cut-off values were generated through ROC analysis of 341 serum samples for *Borrelia* specific IgG **(A-F)** or IgM **(G, H)**. The categorization of the samples into *Borrelia* negative and positive was based on the measurement of the samples with the recomBead Borrelia IgG/IgM 2.0 (Mikrogen). For each antigen, the true positive rate (Sensitivity %) was plotted against the false positive rate (100 % - Specificity %) and the AUC values were calculated.


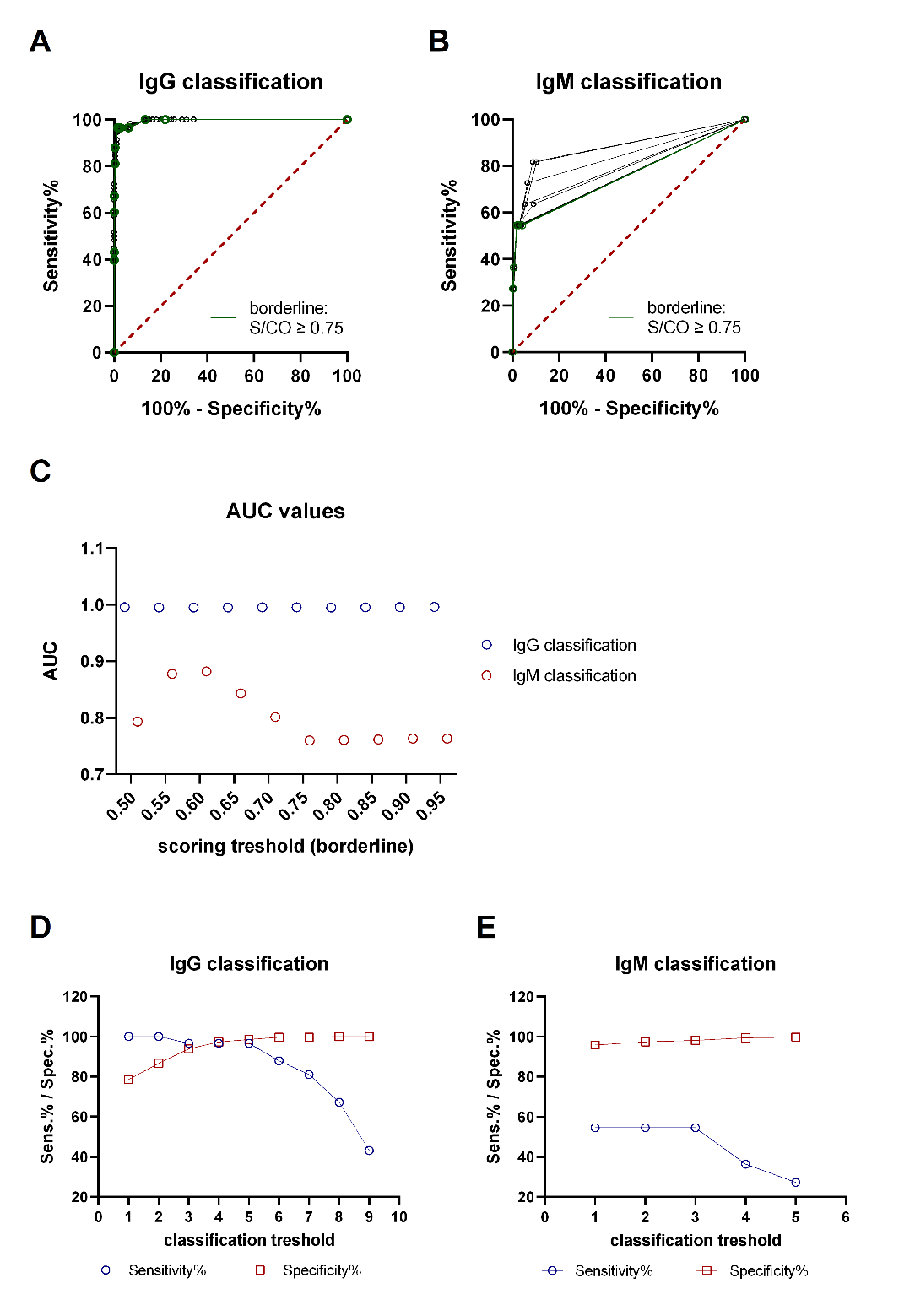


**Figure S3: Sample classification algorithms optimisation**

For sample classification, a scoring system dividing the calculated S/CO values into three categories (negative, borderline and positive) was utilized. While a S/CO >1 was used for the positive samples (due to generation of CO samples), different borderline values were assessed in a step-wise fashion from 0.5 to 1.0 (**A-C**). Assay performance for each borderline value was assessed by ROC analysis, with 0.75 identified as the optimum value. The Sum of Points value for final sample classification was also evaluated (**D-E**), with 5 and 3 points for IgG and IgM respectively being the optimal values.

**
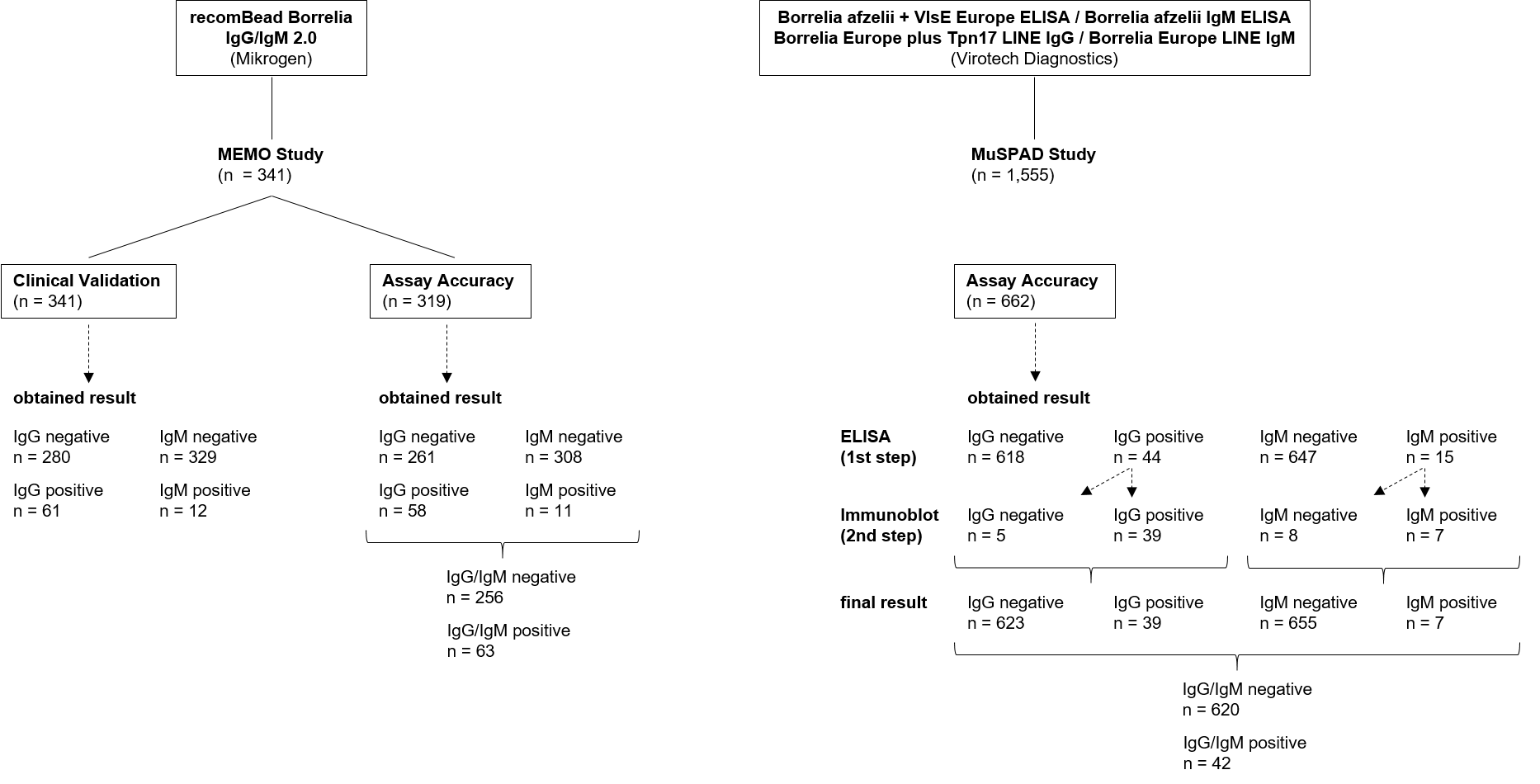
**

**Figure S4: Sample analyses with commercial *Borrelia* tests.**  In addition to the Borrelia multiplex, commercially available *Borrelia* tests were used for the clinical validation or the determination of the assay accuracy. Samples from the MEMO study were measured with the bead-based immunoassay recomBead Borrelia IgG/IgM 2.0. Samples from the MuSPAD study were measured with a recommended 2-step diagnostics consisting of an ELISA and an Immunoblot.

**Figure S5: Comparison of PE detection system on FLEXMAP 3D and INTELLIFLEX platform.** Serum samples (n = 4) were measured for *Borrelia* specific IgG **(A-E)** or IgM **(F)** using PE conjugated species-specific detection antibodies. MFI signals for the individual IgG or IgM dominant antigens were plotted against each other and analyzed by linear regression. A linear curve (x = y) shown as red dashed line indicates identical MFI signals for both platforms. To assess the correlation, Spearman analysis was performed with the r value provided.

**Figure S6: Comparison of IgG detection systems on INTELLIFLEX platform.** Serum samples (n = 12) were measured for *Borrelia* specific IgG using either a PE conjugated or a biotinylated species-specific detection antibody. The biotinylated antibody was used in combination with a BV421 labelled streptavidin. MFI signals for the individual IgG dominant antigens were plotted against each other and analyzed by linear regression. A linear curve (x = y) shown as red dashed line indicates identical MFI signals for detection systems. To assess correlation, Spearman analysis was performed with the r value provided.

**Figure S7: Correlation between single detection and dual detection of IgG/IgM antibodies.** *Borrelia* specific IgG and IgM antibodies were detected in serum samples (n = 12) either separately or in combination. The MFI signals for the individual IgG **(A-E)** and IgM **(F)** dominant antigens were plotted against each other and analyzed by linear regression. A dashed red line shows the curve for identical MFI signals. To assess the correlation, Spearman analysis was performed with the r value provided.

**Figure S8: Manual and automated processing of Borrelia multiplex.** 662 serum samples were measured with the Borrelia multiplex and a common 2-step diagnostics. For the Borrelia multiplex processing was either manually or by pipetting robot. **(A-F)** Correlation between manual and automated processing of Borrelia multiplex. S/CO values were plotted against each other. A red dashed line shows the curve for identical S/CO values. Spearman correlation analysis was performed, for which the r are provided. **(G-R)** Box-Whisker plots of S/CO values for Borrelia negative and positive samples. Samples were categorized according to the result of the 2-step diagnostics. Boxes include the median and the 25th and 75th percentiles. Whiskers are limited to 1.5 times IQR. Outliers are shown as depicted circles. A dashed line (S/CO = 1.0) indicates the threshold between positive and negative signal. The grey area below the line gives the borderline area (0.75 < S/Co < 1.0). For statistical analysis Mann-Whitney test (two-tailed) was used. P values were classified as follows: > 0.05 (ns), ≤ 0.05 (*), ≤ 0.01 (**), ≤ 0.001 (***).
